# Deep Genotypic Species Delimitation of *Aspergillus* Section *Flavi* Isolated from Brazilian Foodstuffs and the Description of *Aspergillus annui* sp. nov. and *Aspergillus saccharicola* sp. nov.

**DOI:** 10.3390/jof8121279

**Published:** 2022-12-06

**Authors:** Josué J. Silva, Maria H. P. Fungaro, Xinhui Wang, Thomas O. Larsen, Jens C. Frisvad, Marta H. Taniwaki, Beatriz T. Iamanaka

**Affiliations:** 1 Centro de Ciência e Qualidade de Alimentos, Instituto de Tecnologia de Alimentos, Campinas 13070-178, São Paulo, Brazil; 2 Centro de Ciências Biológicas, Universidade Estadual de Londrina, Londrina 86057-970, Paraná, Brazil; 3 Department of Biotechnology and Biomedicine, DTU-Bioengineering, Technical University of Denmark, 2800 Kongens Lyngby, Denmark

**Keywords:** coalescence-based, genealogical concordance, phylogenetic, genetic diversity, aflatoxin, taxonomy

## Abstract

*Aspergillus* section *Flavi* is a fungal group that is important in food because it contains spoilage and potentially aflatoxigenic species. Aflatoxins are metabolites that are harmful to human and animal health and have been recognized as the primary natural contaminant in food. Therefore, recognizing the biodiversity of this group in food is necessary to reduce risks to public health. Our study aimed to investigate the diversity of *Aspergillus* section *Flavi* isolated from Brazilian foodstuffs such as cassava, sugarcane, black pepper, paprika, Brazil nuts, yerba-mate, peanuts, rice, and corn. A polyphasic approach integrating phenotypic data and multilocus genotypic analyses (*CaM*, *BenA,* and *RPB2*) was performed for 396 strains. Two new species in the *Aspergillus* subgenus *Circumdati* section *Flavi* are proposed using maximum-likelihood analysis, Bayesian inference, and coalescence-based methods: *Aspergillus saccharicola* sp. nov. and *Aspergillus annui* sp. nov. *A. saccharicola* sp. nov. belongs to the series *Flavi*, is a potentially aflatoxigenic species (B1, B2, G1, and G2), closely related to *Aspergillus arachidicola*, and was found mostly in sugarcane. *A. annui* sp. nov. was isolated from samples of sweet paprika. To accommodate *A. annui* sp. nov., a new series *Annuorum* was proposed.

## 1. Introduction

Mycotoxins are secondary metabolites produced by a variety of fungal species that colonize different crops around the world. More than 400 mycotoxins have been described, but only a few have relevance as food contaminants [1]. In addition to the risk to public health, mycotoxin contamination causes losses that can be estimated in billions of dollars [2]. According to the annual report of the Rapid Alert System for Food and Feed (RASFF), mycotoxins were the main hazard in notifications of border rejection in the European Union in 2020, with nuts, nut products, seeds, fruits, and vegetables accounting for more than 75% of notifications related to mycotoxins [3].

Aflatoxin contamination has been reported primarily in peanuts, corn, spices, nuts (almonds, pistachios, hazelnuts, pecan nuts, and Brazil nuts), fruits, vegetables, and milk [4]. The International Agency for Research on Cancer (IARC) has classified aflatoxins (AFB1, AFB2, AFG1, AFG2, AFM1, and AFM2) in Group 1 as carcinogenic to humans [5]. Furthermore, exposure to these aflatoxins has been linked to immunosuppression, hepatotoxicity, and other pathological effects [6,7].

*Aspergillus* section *Flavi* is a filamentous fungi group that is extremely important in foods and is one of the most concerning issues for agribusiness and industry, as it harbors several potentially toxigenic species. The most important mycotoxins produced by this group are aflatoxins (types B and G) and ochratoxin A (OTA). *Aspergillus* section *Flavi* harbors most of the aflatoxin-producing species [8], with 18 species in this group recognized as aflatoxigenic [9].

OTA is a mycotoxin produced by several species of the genera *Aspergillus* and *Penicillium*. The IARC classified OTA as possibly carcinogenic to humans, in group 2B [8]. Furthermore, OTA has nephrotoxic, hepatotoxic, immunotoxic, and neurotoxic activity. It has resistance to heat and can withstand temperatures of up to 250 °C, preventing its complete elimination in cooking and roasting processes [10,11]. The occurrence of OTA has been reported in cereals (corn, oats, barley, rice, and wheat) [12,13,14,15], nuts and peanuts [16], beer, grape juice, wine and grapes [17,18,19,20], coffee beans [21], cocoa [22,23], spices [24], meat products [25,26], cattle feedstuffs, and dairy products [27,28].

Among the *Aspergillus*, *A*. section *Circumdati* contains the most ochratoxigenic species [8]. Four species in *Aspergillus* section *Flavi* can produce OTA, i.e., *A. alliaceus*, *A. vandermerwei*, *A. neoalliaceus*, and *A. magaliesburgensis*, all belonging to the ser. *Alliacei* [9].

The traditional taxonomy of *Aspergillus* section *Flavi* is based on phenotypic characters (physiological and morphological characteristics), which severely limited its resolution power due to the limited amount of information available to characterize the similarities and differences. In the last decade, *Aspergillus* taxonomy has evolved toward a broader approach (polyphasic taxonomy) that integrates phenotypic (morphology and secondary metabolites) and genotypic (molecular markers) data [29].

The use of genotypic data enabled a larger number of comparable characters. Consequently, greater power of discrimination, the dissemination of molecular techniques, and phylogenetic analysis contributed to a more accurate and dynamic taxonomy. Thus, the inclusion of new species, and the exclusion of other species as a result of synonymy recognition, became more frequent.

Frisvad et al. [9] recently reviewed the taxonomy of *Aspergillus* section *Flavi* using a polyphasic approach, with emphasis on Genealogical Concordance Phylogenetic Species Recognition (GCPSR), a technique that compares the genealogy of different loci. The authors analyzed the nucleotide sequences of the *BenA*, *CaM*, and *RPB2* loci of approximately 200 strains of *Aspergillus* section *Flavi* to identify more clearly the species boundaries. Furthermore, the taxonomic context of the group was significantly changed through the description of new species, dismemberment, synonymization, and renaming [9]. This new taxonomic status of *Aspergillus* section *Flavi* would have an impact on new research and may also influence conclusions from previously reported work through data reinterpretation, generating new understandings.

Brazil is the fourth-largest producer and second-largest exporter of grain in the world [30]. Additionally, it is a leader in the production of beef and one of the top 10 largest producers of vegetables, fruits, nuts (Brazilian nuts and walnuts), and numerous other agricultural and livestock products [31], making this country one of the biggest players in the global food supply chain.

Therefore, the objective of our study was to clarify the biodiversity of *Aspergillus* section *Flavi* isolated from different foodstuffs produced in Brazil, under the perspective of the new taxonomic status of the group, along with genealogical concordance and coalescence-based methods.

## 2. Materials and Methods

### 2.1. Taxon Sampling and Molecular Methods

A total of 396 strains were obtained from the collections of the Institute of Food Technology Institute (ITAL, São Paulo, Brazil) and the Laboratory of Molecular Biology of Fungi at the Londrina State University (UEL, Paraná, Brazil). The strains were isolated from foods collected in different regions of Brazil and were previously identified as belonging to *Aspergillus* section *Flavi* morphologically. Strains were isolated from sugarcane (*n* = 45), corn (*n* = 39), Brazil nuts (*n* = 30), yerba-mate (*n* = 47), peanuts (*n* = 77), rice (*n* = 38), cassava (*n* = 48), black pepper (*n* = 30), and paprika (*n* = 42).

The strains were purified by monosporic culture and cultivated in yeast extract sucrose medium (liquid) at 25 °C for 3 days until the formation of a mycelial film was formed. Later, the mycelia were removed from the culture medium and macerated with liquid nitrogen. The NucleoSpin Microbial DNA kit (Macherey-Nagel, Düren, NRW, Germany) was used to extract genomic DNA according to the protocol recommended by the manufacturer. DNA was quantified spectrophotometrically using NanoDropTM 2000/2000c (Thermo Fisher Scientific, Pittsburgh, PA, USA).

Three-loci amplification was performed via PCR; part of the gene encoding calmodulin (C*aM*) was amplified using the CMD5 and CMD6 [32] or CF1 and CF4 [33] primer pairs; Bt2a and Bt2b primer pair [34] were used for amplification of part of the beta-tubulin gene (*BenA*), and finally, for amplification of the gene of the second largest subunit of RNA polymerase II (*RPB2*), the 5Feur and 7CReur primer pair [35] was used. Conditions for amplification were as described by Silva et al. [36].

The amplification products were separated using agarose gel (1% *w*/*v*) electrophoresis, stained with ethidium bromide, and photographed under ultraviolet light. The PCR products were purified with the ExoSAP-IT™ PCR Product Cleanup reagent (Thermo Fisher Scientific, Santa Clara, CA, USA) after amplification. The PCR fragments were subjected to direct sequencing using the method described by Sanger et al. [37]. Amplicons were sequenced in both directions (forward and reverse) using a BigDye^®^ Terminator v3.1 Cycle Sequencing Kit (Applied Biosystems, Foster City, CA, USA) in a SeqStudio Genetic Analyzer^®^ (Applied Biosystems, Waltham, MA, USA).

### 2.2. Phylogenetic Analysis

The partial sequences of the genes *BenA*, *CaM*, and *RPB2* were used to analyze the diversity of *Aspergillus* section *Flavi* isolates. ClustalW was employed to align the sequences [38] in the BioEdit Sequence Alignment Editor v.7.1.3.0 software [39]. The three loci were concatenated for each of the 396 strains, and haplotype analysis was performed using the DnaSP v.6 DNA Sequence Polymorphism program [40].

Furthermore, maximum-likelihood (ML) trees were constructed using a representative of each of the characterized haplotypes to identify the strains. Phylogenetic trees were constructed for each of the loci separately, and for the combined dataset (*CaM+BenA+RPB2*). The sequences were aligned with those of type or neotype strains of species formally accepted in the *Aspergillus* section *Flavi*, using ClustalW.

For the construction of ML trees, the best nucleotide substitution model was calculated in the jModelTest2 [41], based on the Akaike Information Criterion (AIC). The ML trees were built in the MEGA 11 program [42], with 1000 bootstraps of replicates.

Additionally, descriptive DNA parameters such as nucleotide diversity (π), number of variables sites, and parsimony-informative sites were measured for each of the three loci individually and for the combined dataset using the DnaSP v.6 DNA Sequence Polymorphism program.

All intra- and interspecific variability (based on haplotypes) discovered in this study were deposited in GenBank, and accession numbers can be found in the Supplementary Material (Table S1).

### 2.3. Coalescence Analysis

For the coalescence analysis, in addition to the ML trees, we also performed Bayesian inference (BI) analysis for each of the three loci individually. The BI analysis was conducted in MrBayes 3.2.3. The most suitable nucleotide substitution model for each dataset was selected based on the lowest Bayesian Information Criterion (BIC) value in jModeltest2. For BI, the Markov Chain Monte Carlo (MCMC) algorithm was run for 5 × 10^6^ generations with a sample frequency of 100 and with 25% of trees removed for burn-in. Convergence diagnostics were monitored based on standard deviations of frequencies below 0.01.

All of the ML and BI gene trees generated with their bootstraps and posterior probabilities scores were used as the input for the program Astral-II [43] in the CIPRES science gateway [44]. The Astral analysis was performed with the full annotation mode, greedy resolution, and 10^6^ replicates for each locus. All other parameters were set to default values. The species tree was visualized using MEGA 11.

### 2.4. Genetic Distance Analysis

We selected reference sequences of *Aspergillus* section *Flavi* species from GenBank PopSet’s (*CaM-*145573188-157381153-158515852-1455806316-1735344820-257480544; *BenA-*133741551-157381072-158144689-257480522-1455803795-1735344018 and *RPB2*-158138946-158144533-372120727-1455806002-1735343742), together with sequences of candidate species of the present study. A pairwise ρ-distance matrix was calculated in the MEGA 11 program using the combined dataset (*CaM+BenA+RPB2)*. The datasets used are available in the Supplementary Material (Table S2).

### 2.5. Morphological Analysis

Morphological analyses were performed according to the recommendations of Samson et al. [29]. For macromorphological observations, representatives of candidate species (groups 1 and 2) were inoculated at three points on Czapek Yeast Autolysate agar (CYA), malt extract agar (MEA), yeast extract agar Sucrose Agar (YESA), and incubated for 7 days at 25 °C in the dark. Furthermore, the strains were tested in CYA medium for 7 days at temperatures of 37 °C and 42 °C. The experiments were performed in triplicate.

Microscopic mounts were made in lactic acid from colonies of MEA (7 days at 25 °C) for micromorphological analysis (optical microscopy). The size of the microstructures: conidiophores, stipes, vesicles, conidia, metulae, and phialides were measured using the software Carl Zeiss™ AxioVision Release 4.8.2.

### 2.6. Secondary Metabolite and Mycotoxin Analysis

Secondary metabolites and mycotoxins were detected in three isolates from each of the new taxa. The six isolates were three points inoculated on CYA, YESA, WATM (Wickerham’s Antibiotic Test Medium) [45], and *Aspergillus flavus parasiticus* agar (AFPA) [46] and incubated at 25 °C for 7 days in the dark. One extract was made from three agar plugs each of CYA and YESA, and another extract was made from three agar plugs of WATM and three agar plugs of AFPA. The extraction solvent used was ethyl acetate isopropanol (3:1, *vol*/*vol*) containing 1% formic acid. The extraction procedure and HPLC (high performance liquid chromatography) analysis was carried out as described by Nielsen et al. [47]. The extracts were subjected to HPLC-DAD and HPLC-DAD-MS-MS (high-performance liquid chromatography–diode array detection-mass spectrometry-mass spectrometry), as described by Nielsen et al. [47] and Wang et al. [48]. The identity was verified by comparing the results to authentic standards (retention time, retention index, UV spectra, and mass spectra).

Ultra-high-performance liquid chromatography–diode array detection–quadrupole time-of-flight mass spectrometry (UHPLC–DAD–QTOFMS) was performed on an Agilent Infinity 1290 UHPLC system (Agilent Technologies, Santa Clara, CA, USA) equipped with a diode array detector. Separation was achieved on a 150 mm × 2.1 mm i.d., 1.9 µm, Poroshell 120 Phenyl Hexyl column (Agilent Technologies, Santa Clara, CA, USA) held at 40 °C. The sample, 2 µL, was eluted at a flow rate of 0.35 mL min^−1^ using a linear gradient from 10% acetonitrile (LC-MS grade) in Milli-Q water buffered with 20 mM formic acid increasing to 100% in 15 min, staying there for 2 min before returning to 10% in 0.1 min. Starting conditions were held for 2 min before the following run.

Mass spectrometry (MS) detection was performed on an Agilent 6545 QTOF MS equipped with Agilent Dual Jet Stream electrospray ion source (ESI) with a drying gas temperature of 250 °C, a gas flow of 8 L min^−1^, sheath gas temperature of 300 °C, and flow of 12 L min^−1^. Capillary voltage was set to 4000 V and nozzle voltage to 500 V in positive mode. MS spectra were recorded as centroid data, at an *m/z* of 100–1700, and auto MS/HRMS fragmentation was performed at three collision energies (10, 20, and 40 eV) on the three most intense precursor peaks per cycle. The acquisition rate was 10 spectra s-1. Data were handled using Agilent MassHunter Qualitative Analysis software (Agilent Technologies, Santa Clara, CA, USA). Lock mass solution in 70% MeOH in water was infused in the second sprayer using an extra LC pump at a flow of 15 μL/min using a 1:100 splitter. The solution contained 1 μM tributylamine (Sigma-Aldrich) and 10 μM Hexakis (2, 2, 3, 3-tetrafluoropropoxy) phosphazene (Apollo Scientific Ltd., Cheshire, UK) as lock masses. The [M + H]+ ions (*m/z* 186.2216 and 922.0098, respectively) of both compounds were used. 

An in-house fungal metabolite library search was carried out as described by Kildgaard et al. [49]. Data files were processed in MassHunter workstation B.07.00 with “Find by Auto MS/MS function” with a processing limit to 200 largest peaks and mass match tolerance *m/z* 0.05. The HRMS/MS library search was performed using a parent and fragment ion accuracy of 20 ppm + 2 mDa, with a minimal forward score of 50 and reverse score of 80.

## 3. Results and Discussion

### 3.1. Genotypic Analysis

Primarily, it is important to contextualize the taxonomic status of *Aspergillus* section *Flavi*; Frisvad et al. [9] carried out the most recent major review of the group, with the authors including seven new species: *Aspergillus aflatoxiformans*, *Aspergillus aspearensis*, *Aspergillus austwickii*, *Aspergillus neoalliaceus*, *Aspergillus subflavus*, *Aspergillus pipericola*, and *Aspergillus vandermerwei*. The authors also synonymized *Aspergillus parvisclerotigenus* and *Aspergillus subolivaceus* as *A. flavus* and *Aspergillus albertensis* as *A. alliaceus*. The other significant points were the renaming of *Aspergillus korhogoensis* to *Aspergillus cerealis* and *Aspergillus bombycis* to *Aspergillus luteovirescens*. Concluding the review, *Aspergillus* section *Flavi* harbored 33 species.

The taxonomy of this group has been dynamic, and the popularization of multiloci analysis associated with genealogical concordance has increased the number of descriptions of new species. After the review by Frisvad et al. [9], six new species of *Aspergillus* section *Flavi* have been proposed—*Aspergillus texensis* [50], *Aspergillus agricola* and *Aspergillus toxicus* [51], *Aspergillus krugeri* and *Aspergillus magaliesburgensis* [52], and more recently, *Aspergillus burnetii* [53].

The three loci analyzed here (*CaM*, *BenA*, and *RPB2*) are considered secondary barcodes of *Aspergillus*, as the official fungal barcode (ITS region) does not have discriminatory power for many *Aspergillus* species [29]. Based on the combined dataset (*CaM*+*BenA*+*RPB2*), 48 haplotypes were characterized; the distribution of haplotypes and the origin of the strains are provided in the Supplementary Material (Table S1). The best nucleotide substitution model was calculated based on jModelTest2 and is presented in Table 1.

**Figure 1:**
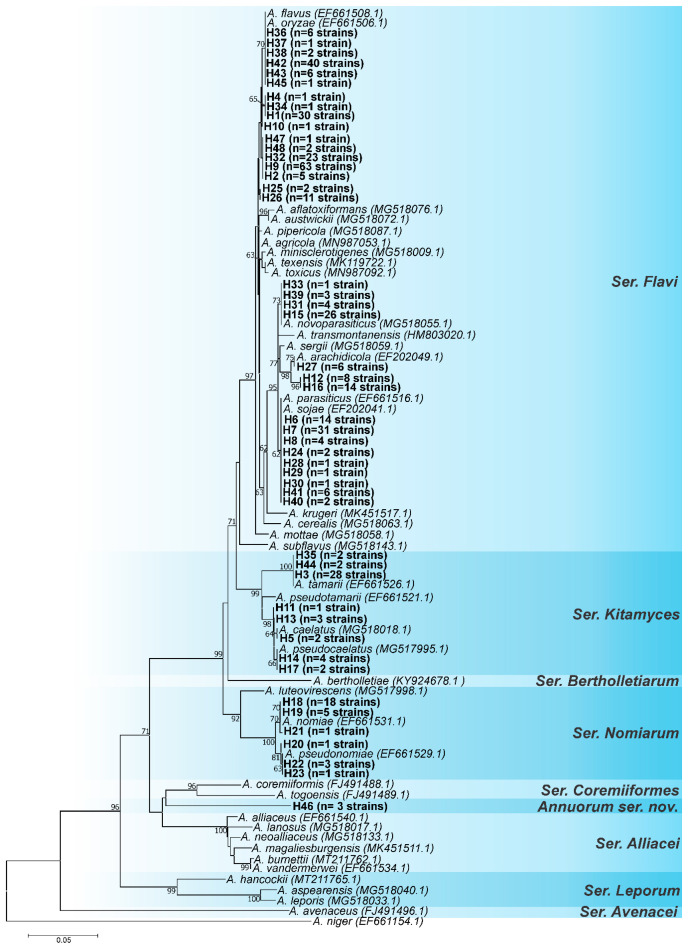
Maximum-likelihood tree of *Aspergillus* section *Flavi* based on *CaM* sequences. Haplotypes obtained in this study are indicated by the letter H in bold. Only bootstraps ≥ 60% are shown. *Aspergillus niger is* the outgroup.

**Figure 2:**
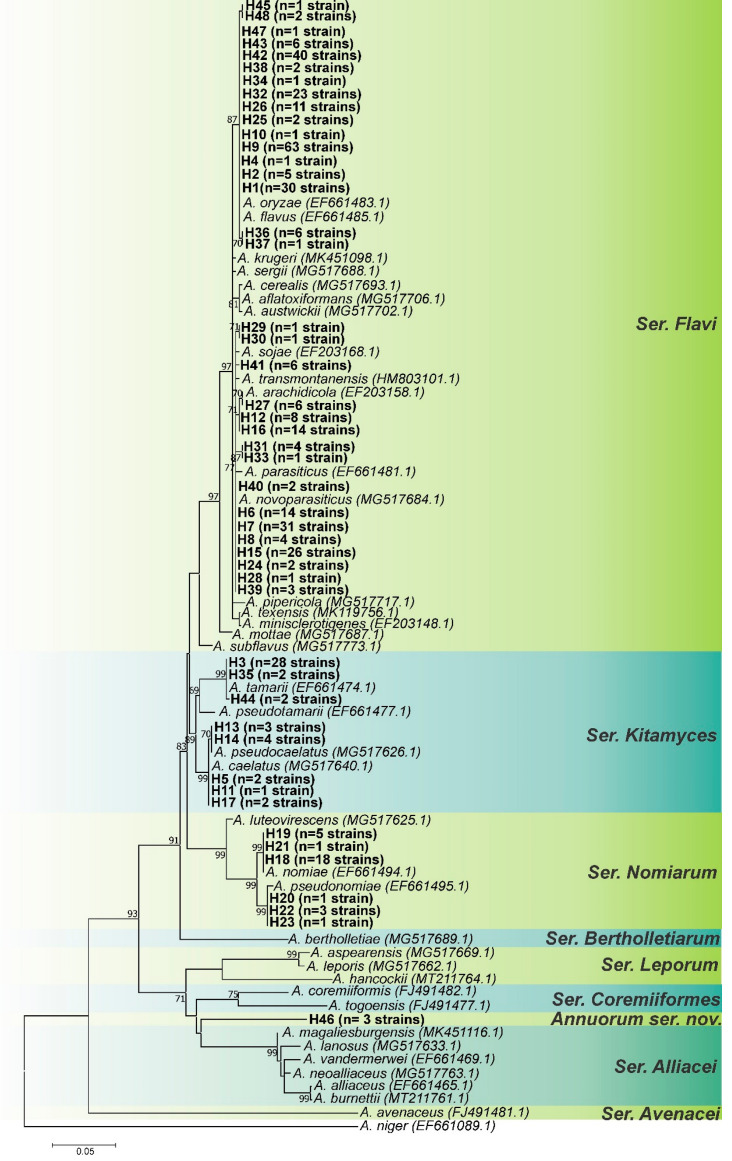
Maximum-likelihood tree of *Aspergillus* section *Flavi* based on *BenA* sequences. Haplotypes obtained in this study are indicated by the letter H in bold. Only bootstraps ≥ 60% are shown. *Aspergillus niger is* the outgroup.

**Figure 3:**
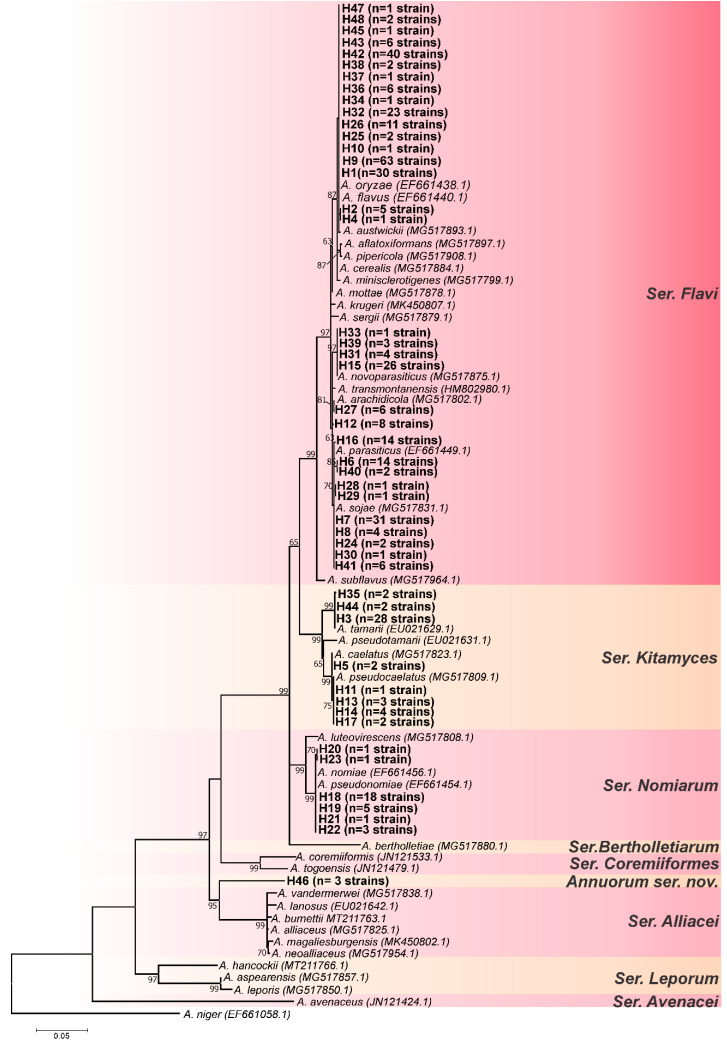
Maximum-likelihood tree of *Aspergillus* section *Flavi* based on *RPB2* sequences. Haplotypes obtained in this study are indicated by the letter H in bold. Only bootstraps ≥ 60% are shown. *Aspergillus niger* is the outgroup.

The *RPB2* locus had the highest number of parsimony-informative sites; however, it also had the lowest nucleotide diversity (Table 1). The *CaM* locus had the most polymorphic sites and the highest nucleotide diversity, whereas the *BenA* locus had the lowest values of polymorphic sites and parsimony-informative sites (Table 1). The three loci appeared to have a high potential for resolution, but the use of one or the other should be avoided in some cases. For instance, *A. parasiticus* and *A. novoparasiticus* are not well discriminated by the *BenA* locus [9]. Based on the tree topologies of *CaM* (Figure 1), *BenA* (Figure 2), and *RPB2* (Figure 3) loci, the *CaM* locus appears to be the best at resolving the *Aspergillus* section *Flavi* species.

Aime et al. [54] recently published a detailed guide on the requirement to describe new fungal species according to the International Code of Nomenclature for algae, fungi, and plants (ICNafp); one of the points highlighted by the authors is the sampling, both analyzed strains and loci. The use of a single locus for the description of a new species is strongly discouraged, but in exceptional cases, it may be acceptable.

The use of multiple unlinked loci increases informativeness and decreases bias caused by combined selection pressure on a single region of the genome, resulting in more reliable evolutionary hypotheses [55,56]. Aime et al. [54] suggest that working with multiple loci is ideal, and using genealogical concordance in the delimitation of cryptic species should be used whenever possible.

More broadly, the concept of genealogical concordance has been used to support the description of many fungal species [9,36,57,58,59]. However, we emphasize that the delimitation of phylogenetic species provided by the genealogical concordance must serve as taxonomic hypotheses, and therefore, must be tested with additional data through integrative taxonomy.

Another fundamental point for our study was the understanding of the concept of phylogenetic species, which is generally supported by the monophyly criterion, which states that species is defined as the smallest group of organisms that includes the common ancestor and all its descendants [60].

In an attempt to improve the recognition of phylogenetic species’ boundaries, Taylor et al. [61] and Dettman et al. [62] developed a theoretical-operational protocol, which they called Genealogical Concordance Phylogenetic Species Recognition (GCPSR). This method employs the use of multiple independent genes, for which gene trees are constructed and compared separately. When using the GCPSR, the subjectivity of recognizing the limits of a species can be reduced, since it is sufficient to look for the point at which there was a transition from concordance between gene trees (representing the divergent phylogenetic relationships of the species tree) to the incongruity between gene trees (representing the cross-linked associations between individuals within a species). In other words, the intersection of conflicting topologies indicates gene flow between individuals below the level of species (intraspecies).

**Figure 4:**
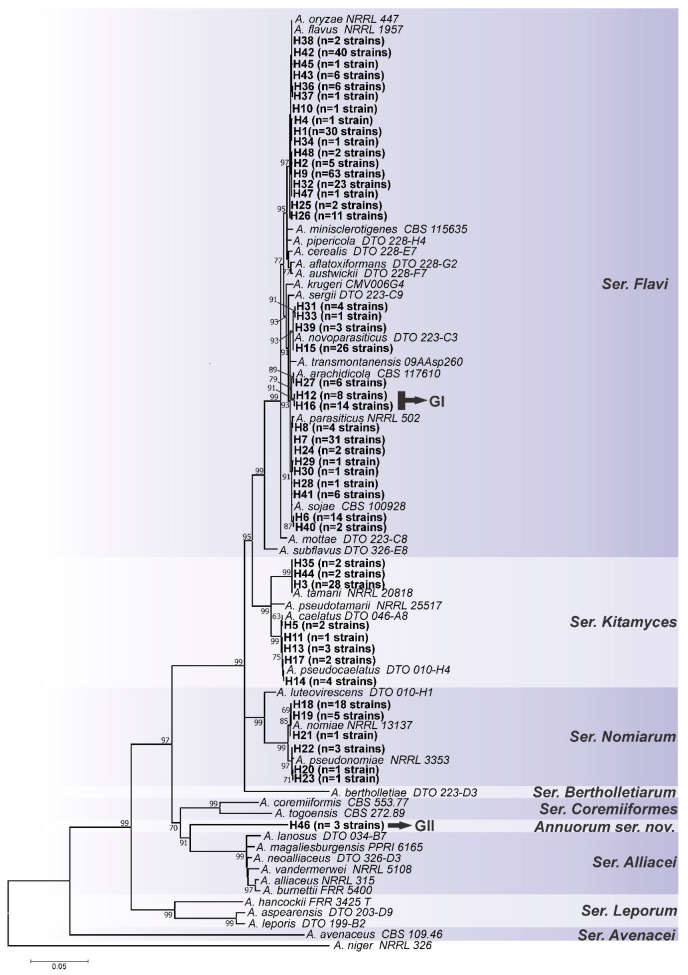
Maximum-likelihood tree of *Aspergillus* section *Flavi* based on combined dataset sequences (*CaM+BenA+RPB2*). Haplotypes obtained in this study are indicated by the letter H in bold. Only bootstraps ≥ 60% are shown. *Aspergillus niger* is the outgroup.

Additionally, the analysis must be conducted with a good sampling of strains, as the description of a new species based on a single strain is not recommended. The evaluation of multiple strains allows for a more comprehensive view of the genetic composition of the group under analysis [63]. We used genealogical concordance analysis to 1188 nucleotide sequences obtained from 396 strains isolated from 9 foodstuffs to investigate the context of *Aspergillus* section *Flavi* species in Brazilian foodstuffs,

According to this analysis, as can be seen in Figure 1, Figure 2, Figure 3 and Figure 4, the most common species is undoubtedly *A. flavus*, with 194 strains distributed in 17 haplotypes (H1, H2, H4, H9, H10, H25, H26, H32, H34, H36, H37, H38, H42, H43, H45, H47, H48), followed by *A. parasiticus* with 63 individuals, distributed in 9 haplotypes (H6, H7, H8, H24, H28, H29, H30, H40, H41), and together these two species represented 64.2% of the total set of strains. This is not a surprise as *A. flavus* and *A. parasiticus* are considered the most frequent species of *Aspergillus* section *Flavi* and the most important from the perspective of aflatoxin production [64]. Other species found were: *A. novoparasiticus*, *A. arachidicola*, *A. tamarii*, *A. caelatus*, *A. pseudocaelatus*, *A. nomiae*, and *A. pseudonomiae* (Figure 4).

The *CaM* ML tree (Figure 1) allowed for the allocation of all haplotypes together with species formally accepted in *Aspergillus* section *Flavi*, except for haplotypes H12 and H16 (closer to *A. arachidicola*); H46 (intermediate to ser. *Coremiiformes* and ser. *Alliacei*); and H25 and H26 (closer to *A. flavus/oryzae*).

Moreover, the formation of subgroups within the *A. flavus/oryzae* clade is also visible (Figure 1); *A. flavus/oryzae* exhibits high intraspecific diversity, and the formation of these subgroups is common. Some of these subgroups correspond to the type strains of *A. kambarensis* (CBS 542.69) and *A. subolivaceus* (CBS 501.65), both of which are considered synonymous with *A. flavus sensu stricto*.

The *BenA* locus exhibits less intraspecific variation for members of the *A. flavus/oryzae* group, resulting in a less branched topology; haplotypes H25 and H26 were grouped together with type strains of *A. flavus* and *A. oryzae* (Figure 2).

The *BenA* ML tree (Figure 2) did not allow for a good resolution of the species of the *A. parasiticus* group (*A. arachidicola*, *A. parasiticus* and *A. novoparasiticus*). As already mentioned, the *BenA* locus does not lend itself to discrimination between *A. parasiticus* and *A. novoparasiticus* [9]. The H12 and H16 haplotypes, again, did not fully group the *A. arachidicola*-type strain (CBS 117610).

The *BenA* locus also did not allow for the grouping of Haplotype H46 to any species of *Aspergillus* section *Flavi* (Figure 2); again, a distinct clade was formed between ser. *Coremiiformes* and ser. *Alliacei*, but phylogenetically closer to ser. *Alliacei*.

As in the *CaM* and *BenA* ML trees, for the *RPB2* locus (Figure 3), the haplotype H46 also formed a distinct clade from the others, more phylogenetically related to being ser. *Alliacei*. The same occurred in the concatenated ML tree (Group II) (Figure 4). This topological concordance at different loci evidences an independent evolutionary lineage [61,62], thus, H46 shows reciprocal monophyly in relation to all species formally accepted in *Aspergillus* section *Flavi*. Therefore, based on the concept of phylogenetic/genealogical species, we started to treat it as a new species in *Aspergillus* section *Flavi*, entitled *Aspergillus annui* sp. nov.

Similarly to what occurs for *A. parasiticus* and *A. novoparsiticus* with the locus *BenA*, using the *RPB2* locus for identification or discrimination between *A. nomiae* and *A. pseudonomiae* should be avoided, as the difference between the aforementioned species for the *RPB2* locus is only one snip, which is in a marginal portion, and is easily lost in alignments of large datasets.

Interestingly, the *RPB2* ML tree genealogy showed discordance with *CaM* and *BenA* for the H12 and H16 haplotypes that clustered together with *A. parasiticus/A. sojae* (Figure 3) and did not form a distinct group (closer to *A. arachidicola*), as observed in the other two loci (Figure 1 and Figure 2) and on the concatenated ML tree (group I) (Figure 4).

Similarly, in the ser. *Kitamyces*, the haplotypes H11, H13, and H17 had variable taxonomic positions (Figure 1, Figure 2 and Figure 3), clustering together with *A. caelatus* or *A. pseudocaelatus*, depending on the locus analyzed. This incongruence between gene trees is frequently used to indicate species delimitation, and it is even one of the bases of the GCPSR mentioned above (when caused by recombination). However, it can also be an indication of incomplete lineage sorting (ILS) [65,66].

ILS is an evolutionary phenomenon that transcends speciation events and occurs when ancestral gene sequences fail to coalesce, i.e., the persistence of ancestral polymorphisms across speciation events results in incompletely classified loci, which consequently may cluster closely related non-conspecifics, rather than separate them [67,68,69,70], resulting in hemiplasy [71].

The best method for distinguishing between species boundary-breaking signatures from those associated with ILS is to use methods based on the coalescent theory, which relies on the use of multiple gene trees to examine coalescence, thus, producing species trees; such methods have been used to overcome this type of difficulty in genealogical approaches to species delimitation [72,73,74,75,76,77] and has been successfully applied to fungi [78,79,80,81,82].

Therefore, we constructed species trees based on the coalescent model to elucidate the taxonomic position of haplotypes H12 and H16 (*clade A. parasiticus*) and H11, H13, and H17 (in the ser. *Kitamyces*). In addition, we also implemented this approach for the haplotype H46 (*A. annui* sp. nov.) as additional evidence.

**Figure 5:**
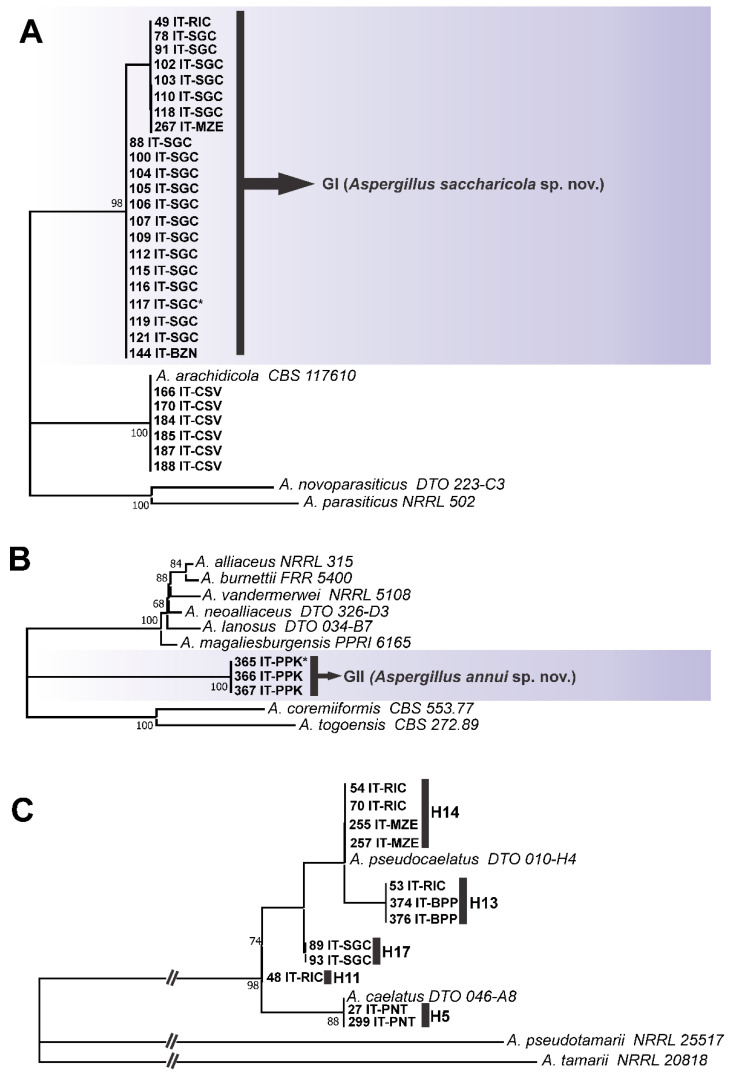
Unrooted coalescent species trees estimated using ASTRAL-II. (**A**) *Aspergillus parasiticus* clade; (**B**) *Alliacei* and *Coremiiformes* series; (**C**) ser. *Kitamyces*. Only bootstraps ≥ 60% are shown. * = type strains of the new species described in this study.

As seen in Figure 5, the set of 22 strains of haplotypes H12 and H16 formed a distinct group of *A. parasiticus*, *A. arachidicola* and *A. novoparasiticus*; GI is statistically well supported. This result shows that in fact GI represents an independent evolutionary lineage, and should be considered a distinct phylogenetic species in *Aspergillus* section *Flavi*, hereinafter referred to as *Aspergillus saccharicola* sp. nov. Furthermore, we constructed ML trees based on other loci (ITS and *niaD*), which also corroborated these results (Supplementary Material Figure S1).

Regarding the GII group (H46), the species tree based on coalescence (Figure 5) corroborates the phylogenetic analyses (Figure 1, Figure 2, Figure 3 and Figure 4) and solidly supports the description of *A. annui* sp. nov.

In the coalescence analysis of the ser. *Kitamyces*, we evaluated 12 strains from five different substrates (maize, black pepper, sugarcane, peanuts, and rice). The haplotypes H11, H13, and H17, that showed incongruences in gene trees, formed a statistically well-supported group (bootstrap 74%) with the type strain of *A. pseudocaelatus* (Figure 5); however, there was still the formation of subgroups, but with little statistical support. This confirms the incongruence between the gene and species trees. It was demonstrated that the ILS can be a substantial phenomenon in the ser. *Kitamyces.*

**Figure 6:**
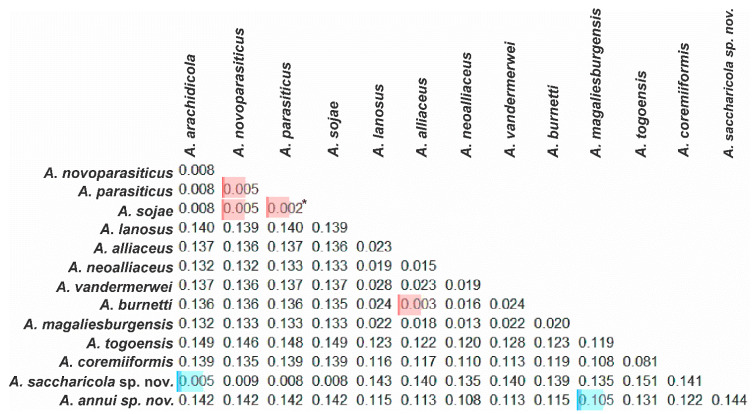
Pairwise genetic distance matrix of the combined dataset (*CaM+BenA+RPB2* sequences), among *Aspergillus* section *Flavi* species. Highlights in red = smallest distances found in this dataset. Highlights in blue = smallest distances found between the newly proposed species *Aspergillus annui* sp. nov. and *Aspergillus saccharicola* sp. nov. in relation to currently accepted species in *Aspergillus* section *Flavi.* * note= *A. sojae* is a non-toxigenic domesticated variant of *A. parasiticus*.

Additionally, we used a dataset of reference sequences (Table S2) to calculate a genetic distance matrix to analyze the divergence between the candidate species of the present study, *A. annui* and *A. saccharicola*, with the species related to them (Figure 6).

The genetic distance between *A. annui* sp. nov. and the species of the series *Alliacei* and *Coremiiformes* was greater than the genetic distance between the species currently accepted in these groups, such as *A. alliaceus* vs. *A. neoalliaceus* (0.015) and *A. togoensis* vs. *A. coremiiformis* (0.081) (Figure 6). Furthermore, the genetic distance of *A. annui* sp. nov. in relation to the individuals of the ser. *Alliacei* and/or ser. *Coremiiformes* was equivalent or greater to that found between the individuals of the ser. *Alliacei* vs. ser. *Coremiiformes* (Figure 6), which denotes the need to establish a new series to accommodate this new species, which was also noticeable in all the genealogies presented (Figure 1, Figure 2, Figure 3, Figure 4 and Figure 5). 

The species with the smallest genetic distance in relation to *A. annui* sp. nov. was *A. magaliesburgensis* (0.105) (Figure 6), a species recently described by Visagie et al. [52].

Considering the members of the *Alliacei* and *Coremiiformes* series, the smallest genetic distance found between the members of these groups was 0.003 (Figure 6), which occurred in the comparison of *A. alliaceus* vs. *A. burnetti*; similar values were obtained in the comparison of *A. parasiticus* and *A. sojae* (0.002).

It is important to note that *A. sojae* is a non-toxigenic domesticated variant of *A. parasiticus*; the same relationship exists between *A. oryzae* and *A. flavus* [83,84,85,86,87]. These species are still classified as separate taxa for economic and food safety reasons, but not for taxonomic reasons. *A. oryzae* and *A. sojae* are widely used in the food industry, and are generally recognized as safe by the FDA [4,86].

The genetic distance of *A. saccharicola* sp. nov. and the closest species (*A. arachidicola*), was also equivalent to that found among other species of the ser. *Flavi*, such as *A. parasicitus* vs. *A. novoparasiticus* (0.005) (Figure 6).

All these results strongly support the description of *A. annui* sp. nov. and *A. saccharicola* sp. nov. based on the concept of phylogenetic/genealogical species; however, as recommended by the ICNafp, we also performed phenotypic analysis.

### 3.2. Phenotypic Analysis

#### 3.2.1. Secondary Metabolites Analysis

*Aspergillus annui* sp. nov. did not produce any mycotoxins on the four media CYA, YESA, WATM, and AFPA, but the metabolites ergokonin B, glycocholic acid, flavin, phytosphingosin, and sphinganin were detected (Table 2). *A. annui* sp. nov. was found to produce the antifungal ergokonin B, which is also produced by *Trichoderma* sp. Xy24 and a *Fusarium* species [88,89]. Ochratoxin A, which is produced by most species in the *Alliacei* series, was not detected in *A. annui* sp. nov.

*Aspergillus saccharicola* sp. nov. produced several known secondary metabolites, including the mycotoxins aflatoxin B1, B2, G1, G2, cyclopiazonic acid, O-methylsterigmatocystin, sterigmatocystin, tenuazonic acid and valine-tenuazonic acid, in addition to aspergillic acid, chrysogine, desertorin A, ergokonin B, kojic acid, nidulanin X6, parasiticolides, and the primary metabolite phytosphingosin (Table 2). 

Moreover, even though sphinganine and phytospingosin are considered as primary metabolites, they may be involved in the biosynthesis of the secondary metabolites flavucides, which are antibacterial cerebrosides known from *A. flavus* [90]. Additionally, flavin is structurally related to the fluorescent secondary metabolites asperopterin A and B, which were isolated from a strain identified as *A. oryzae* [91,92].

The high-resolution MS/MS spectrum of dereplicated metabolites can be found in the Supplementary Material (Table S3).

#### 3.2.2. Morphological Analysis 

To support the description of these new taxa, detailed morphological descriptions and illustrations are provided below.

**Taxonomy**

**Series *Annuorum*** ser. nov. Silva, J.J., Iamanaka, B.T., Frisvad J.C.

**Mycobank**: MB845971

**Etymology**: Named after *Aspergillus annui*

**Type**: *Aspergillus annui* (IBT 36122)

In *Aspergillus* subgen. *Circumdati* sect. *Flavi*.

**Diagnosis**: The *Annuorum* series belongs to the subgenera *Circumdati* in the section *Flavi*. *Annuorum* is a sister group to the *Alliacei* series. There is no growth at 37 °C and 42 °C (CYA, 7d). Sexual morph not observed in culture.

**Included species**: *Aspergillus annui*

**Extrolites**: Ergokonin B, kojic acid, nidulanin X3, nidulanin X5.

**Figure 7:**
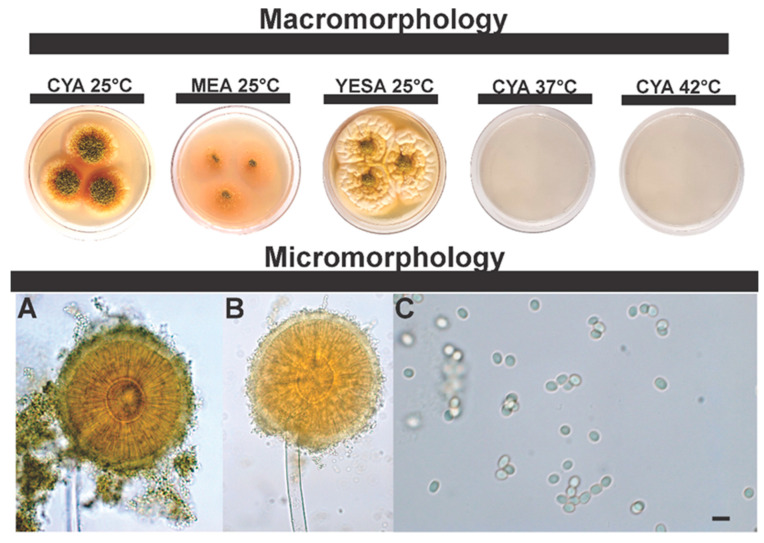
Colonial and microscopic morphology of *Aspergillus annui* sp. nov. (365-IT-PPK = IBT 36122). (**A**,**B**) Conidiophores; (**C**) Conidia (scale bar 5 µm).

***Aspergillus annui* sp. nov.** Silva, J.J., Fungaro M.H.P., Frisvad, J.C., Taniwaki, M.H., Iamanaka, B.T. Figure 7.

**Mycobank**: MB845969

**Etymology**: The specific epithet refers to the substrate from which it was isolated, paprika pepper, *Capsicum annuum*.

In *Aspergillus* subgen. *Circumdati* sect. *Flavi* ser. *Annuorum*.

**Typification**: BRAZIL. São Paulo State, São Paulo City, 23°32′28.6″ S 46°37′44.7″ W, in sweet paprika, 6 April 2017, isolated by Yasumura, C.A., holotype 365-IT-PPK = IBT 36122).

**DNA barcodes**: *BenA* (ON529842), *CaM* (ON529841), *RPB2* (ON529843), ITS (OP691228).

**Colony diam.**: 7 days, 25 °C: CYA 33–37 mm, MEA 33–40 mm, YESA 41–51 mm; CYA 37 °C no growth; CYA 42 °C no growth.

**Diagnosis**: Reduced growth in MEA and CYA (25 °C), does not grow at 37 °C and 42 °C. *Aspergillus annui* is phylogenetically closer to being ser. *Alliacei*, all members of this series grow well in CYA 37 °C. Reduced growth on MEA and CYA (25 °C) media. In CYA (25 °C) there is abundant sporulation in the central region, growing vertically. Ochratoxin A is not produced.

**Colony characters**: On CYA 25 °C, 7 days: Colonies small, grooved; dense green sporulation in the central region, and absence of sporulation on the edges, white to cream colored edges, pinkish soluble pigments, exudates present (colorless), and sclerotia absent. On MEA 25 °C, 7 days: Small colonies, translucent mycelial halo, poor and sparse sporulation, light green. Pigments and sclerotia are absent, exudates transparent, and sporulation in the central region. On YESA 25 °C, 7 days: Colonies moderately deep; cerebriform appearance, low green sporulation in the central region, white edges; soluble pigments, exudates and sclerotia absent.

**Micromorphology**: Conidial heads yellow-green when young, shifting to olive-green in age, biseriate. Conidiophores (88.7 ± 18.7 μm) with smooth stipes, hyaline, 12 ± 2 μm. Metulae, 20.8 ± 4.9 μm; phialides, 6.8 ± 0.9 μm. Vesicles globose to subglobose, 35.5 ± 5.7 μm wide. Conidia smooth to slightly rough, 3.8 ± 0.3 μm. 

**Note**: *A. annui* is phylogenetically closer to *A. magaliesburgensis*, these two species are easily distinguished, either by genotypic or phenotypic data. *A. magaliesburgensis* grows well at 37 °C and produces sclerotia, which are differentiating characteristics from *A. annui*. 

**Figure 8:**
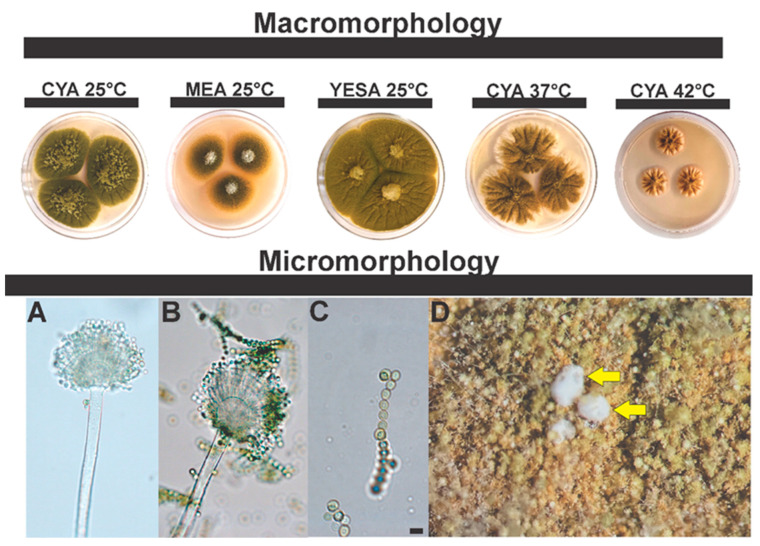
Colonial and microscopic morphology of *Aspergillus saccharicola* sp. nov. (117-IT-SGC = IBT 36126). (**A**,**B**) Conidiophores; (**C**) Conidia (scale bar 5 µm); (**D**) Sclerotia (CYA 37 °C), yellow arrows indicate the structures.

***Aspergillus saccharicola* sp. nov.** Silva, J.J., Frisvad, J.C., Fungaro, M.H.P., Taniwaki, M.H., Iamanaka, B.T. Figure 8.

**Mycobank**: MB845970

**Etymology**: The specific epithet refers to the substrate from which it was isolated, sugarcane, *Saccharum officinarum*.

In *Aspergillus* subgen. *Circumdati* sect. *Flavi* ser. *Flavi*.

**Typification**: BRAZIL. São Paulo State, São Paulo City, 23°35′29.7″ S 46°40′52.1″ W, in sugarcane juice, 14 September 2011, isolated by Iamanaka, B.T, holotype 117-IT-SGC = IBT 36126.

**DNA barcodes**: *BenA* (ON529845), *CaM* (ON529844), *RPB2* (ON529846), ITS (OP611470).

**Colony diam.**: 7 days, 25 °C: CYA 51–59 mm, MEA 40–50 mm, YESA 65–69 mm; CYA 37 °C 42–45 mm; CYA 42 °C 20–22 mm.

**Diagnosis**: Sclerotia production (low but consistent) at CYA 37 °C, white to cream sclerotia, measuring 500–712 µm. At MEA (25 °C). On the MEA medium (25 °C), *A. saccharicola* has a reduced growth pattern compared to *A. arachidicola* (60–65 mm); moreover, colony color and texture are also different in this medium (see description of *A. arachidicola* [93]).

**Colony characters**: On CYA 25 °C, 7 days: Olive green colonies, with aerial mycelium in the central region, abundant sporulation, velvety surface; exudates present (colorless); soluble pigments, and sclerotia absent. On MEA 25 °C, 7 days: Light green colonies, aerial mycelium in the central region. Pigments and sclerotia are absent; transparent exudates. On YESA 25 °C, 7 days: green colonies; velvety appearance; aerial mycelium; soluble pigments, exudates and sclerotia absent.

**Micromorphology**: Conidial heads brown, uniseriate and biseriate. Conidiophores (66.5 ± 10.3 μm) with stipes hyaline, finely roughened, 12.6 ± 1.3 μm. Vesicles globose to subglobose, 30.4 ± 5.3 μm wide. Metulae, 10.4 ± 2 μm; phialides, 5 ± 0.5 μm. Conidia smooth to slightly rough, 4.7 ± 0.6 μm.

## Figures and Tables

**Table 1:** Descriptive parameters of datasets and nucleotide substitution models.

	Substitution Model	Alignment Size	Parsimony-Informative Sites	Polymorphic Sites	Nucleotide Diversity
*CaM*	GTR+GI	474	150	214	0.072
*BenA*	K80+GI	499	139	195	0.071
*RPB2*	GTR+G	622	179	213	0.065
Concatenated data		1595	468	622	0.069

**Table 2:** Production of primary and secondary metabolites, including mycotoxins, by *Aspergillus annui* sp. nov. and *A. saccharicola* sp. nov.

Species	Isolate	Primary Metabolites	Secondary Metabolites
*Aspergillus annui*	365 IT-PPK * = IBT 36122	Glycocholic acid, flavin, pantothenic acid, phytosphingosine, sphinganine	Ergokonin B, kojic acid, nidulanin X3, nidulanin X5
*Aspergillus annui*	366 IT-PPK = IBT 36123	Glycocholic acid, flavin, pantothenic acid, phytosphingosine, sphinganine	Ergokonin B, kojic acid, nidulanin X3, nidulanin X5
*Aspergillus annui*	367 IT-PPK = IBT 36124	Glycocholic acid, flavin, pantothenic acid, phytosphingosine, sphinganine	Ergokonin B, kojic acid, nidulanin X3, nidulanin X5
*Aspergillus saccharicola*	78 IT-SGC = IBT 36125	Phytosphingosine	Aflatoxin B_1_, B_2_, G_1_, G_2_, M_1_, anthranilic acid, aspergillic acid, chrysogine, cyclopiazonic acid, desertorin A, ergokonine B, erythroglaucin, kojic acid, O-methylsterigmatocystin, nidulanin X6, parasiticolide A, sterigmatocystin, tenuazonic acid, valine-tenuazonic acid
*Aspergillus saccharicola*	117 IT-SGC * = IBT 36126	Phytosphingosine	Aflatoxin B_1_, B_2_, G_1_, G_2_, M_1_, anthranilic acid, aspergillic acid, chrysogine, cyclopiazonic acid, desertorin A, ergokonine B, erythroglaucin, kojic acid, O-methylsterigmatocystin, nidulanin X6, parasiticolide A, sterigmatocystin, tenuazonic acid, valine-tenuazonic acid
*Aspergillus saccharicola*	121 IT-SGC = IBT 36127	Phytosphingosine	Aflatoxin B_1_, B_2_, G_1_, G_2_, M_1_, anthranilic acid, aspergillic acid, chrysogine, cyclopiazonic acid, desertorin A, ergokonine B, erythroglaucin, kojic acid, O-methylsterigmatocystin, nidulanin X6, parasiticolide A, sterigmatocystin, tenuazonic acid, valine-tenuazonic acid

* Type strain.

## Data Availability

The sequences newly generated in this study have been submitted to the GenBank database.

## Supplementary Materials

The following supporting information can be downloaded at: https://www.mdpi.com/article/10.3390/jof8121279/s1, Figure S1: Maximum-likelihood tree of *Aspergillus parasiticus* clade based on combined dataset sequences (*ITS+niaD)*; Table S1: Description of the samples from this study. Strains, substrate of origin, haplotypes and GenBank accession numbers; Table S2: Dataset for genetic distance analysis; Table S3: The high-resolution MS/MS spectrum of dereplicated metabolites of *Aspergillus annui* sp. nov. and *Aspergillus saccharicola* sp. nov.
