# Supplementary material for: Deep Genotypic Species Delimitation of Aspergillus Section Flavi Isolated from Brazilian Foodstuffs and the Description of Aspergillus annui sp. nov. and Aspergillus saccharicola sp. nov
Source: J Fungi (Basel). 2022 Dec 6;8(12):1279. doi: 10.3390/jof8121279 (PMC9781283; doi:10.3390/jof8121279)
Supplement: Supplementary file 1 [file jof-08-01279-s001.zip › Figure S1.pdf]

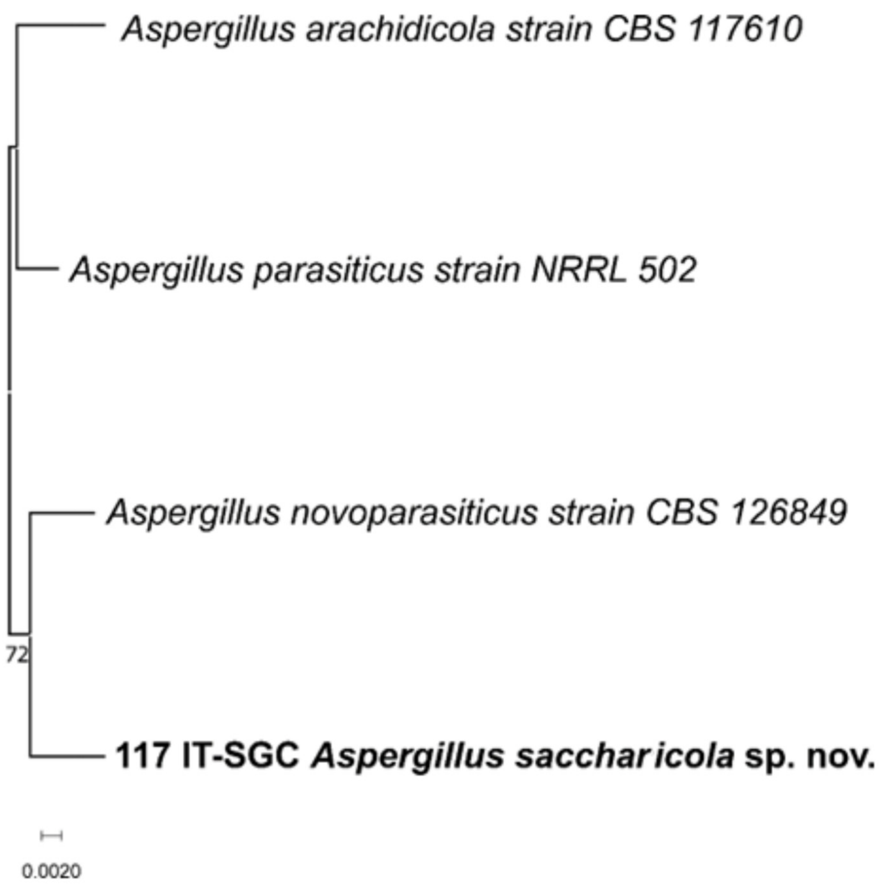

Figure S1. Maximum Likelihood tree of *Aspergillus parasiticus* clade based on combined dataset sequences (*ITS+niaD).*

>117 IT-SGC niaD

ACGGGTGTTCATCCGTTTAACGTTGAGCCACCTCTTACAGATCTGTATAAAGAAGGTACGAGTTATAGC
TGCTCCACCTCCTATCCTCATCAGATTGCTCCCTTGAGCCGTTACATTCTCACACTCTGAAAGGGTTTTTA
ACATCGCCGGAGCTCTTCTATGTTCGAAATCATGGCCCAGTCCCTCATGTCAAGGATGAAGATATCCCTC
ACTGGGAAATTACTATTGAAGGGTTAGTAAAGTACTAGGTTCTCTGCCAAACATCCGCTAACCAAAGTA
TAGACTGGTAGAGAAGCCTTTGGTACTAAACTTCCGACAAGTGTTGCAGCAGTACGACCAAATCACGG
CGCCCATCACCCTTGTGTGTGCAGGCAATCGACGCAAAGAGCAAAACATTGTCCGTAAAACGAAAGGC
TTTTCCTGGGGATCGGCGGGACTATCGACTGCCCTCTTCACTGGCCCATTGCTGGCGGATATCCTCCGC
AGTGCGAAACCCCTGCGTCAAGCGAAATACGTCTGTATGGAAGGAGCGGATAAGCTGGTATGCTGTAC
CTCTATCTTATGATGATAATTACTAAGTTCGCTGCAGCCCAATGGTCACTACGGCACATCTATTAAATTG
AACTGGGCCCT

>117 IT-SGC ITS

GGTTCCTAGCGAGCCCAACCTCCCACCCGTGTTTACTGTACCTTAGTTGCTTCGGCGGGCCCGCCGTCAT
GGCCGCCGGGGGCGTCAGCCCCGGGCCCGCGCCCGCCGGAGACACCACGAACTCTGTCTGATCTAGTG

AAGTCTGAGTTGATTGTATCGCAATCAGTTAAAACTTTCAACAATGGATCTCTTGGTTCCGGCATCGATG
AAGAACGCAGCGAAATGCGATAACTAGTGTGAATTGCAGAATTCCGTGAATCATCGAGTCTTTGAACG
CACATTGCGCCCCCTGGTATTCCGGGGGGCATGCCTGTCCGAGCGTCATTGCTGCCCATCAAGCACGGC
TTGTGTGTTGGGTCGTCGTCCCCTCTCCGGGGGGGACGGGCCCCAAAGGCAGCGGCGGCACCGCGTCC
GATCCTCGAGCGTATGGGGCTTTGTCACCCGCTCTGTAGGCCCGGCCGGCGCTTGCCGAACGCAAAAC
AACCATTCTTTCCAGGTTGACCTCGGATCAGGTAGGGATACCCGC
