# Supplementary material for: Deep Genotypic Species Delimitation of Aspergillus Section Flavi Isolated from Brazilian Foodstuffs and the Description of Aspergillus annui sp. nov. and Aspergillus saccharicola sp. nov
Source: J Fungi (Basel). 2022 Dec 6;8(12):1279. doi: 10.3390/jof8121279 (PMC9781283; doi:10.3390/jof8121279)

Table S3: The high-resolution MS/MS spectrum of dereplicated metabolites of *Aspergillus annui* sp. nov. and *Aspergillus saccharicola* sp. nov

Network clusters	Compounds name	Molecular Formula	m/z	Mass (MFG*)	Species
Aflatoxins	**aflatoxin M1**	$C_{17}O_7H_{12}$	329.0657	328.0583	*A. saccharicola*
	**aflatoxin G2**	$C_{17}O_7H_{14}$	331.0809	330.0739	*A. saccharicola*
	**aflatoxin G1**	$C_{17}O_7H_{12}$	329.0655	328.0586	*A. saccharicola*
	**aflatoxin B2**	$C_{17}O_6H_{14}$	315.0864	314.0790	*A. saccharicola*
	**aflatoxin B1**	$C_{17}O_6H_{12}$	313.0708	312.0633	*A. saccharicola*
	**sterigmatocystin**	$C_{18}O_6H_{12}$	325.0703	324.0633	*A. saccharicola*
	**O-methylsterigmatocystin**	$C_{19}O_6H_{14}$	339.0872	338.0790	*A. saccharicola*
Cyclopiazonic acids	**Cyclopiazonic acid**	$C_{20}N_2O_3H_{20}$	337.1552	336.1480	*A. saccharicola*
Tenuazonic acids	**Tenuazonic acid**	$C_{10}NO_3H_{15}$	198.1128	197.1055	*A. saccharicola*
	**Valine-tenuazonic acid**	$C_9H_{13}NO_3$	184.0964	183.0891	*A. saccharicola*
	**aspergillic acid**	$C_{12}N_2O_2H_{20}$	225.16	224.1527	*A. saccharicola*
	**desertorin A**	$C_{22}H_{18}O_8$	411.1075	410.1002	*A. saccharicola*
	**ergokonin B**	$C_{28}H_{42}O_5$	481.2924	458.3032	*A. saccharicola and A. annui*
	**Nidulanin X3**	$C_{28}H_{36}N_4O_6$	525.2678	524.2634	*A. annui*
	**Nidulanin X5**	$C_{28}H_{36}N_4O_5$	509.2744	508.2685	*A. annui*
	**Nidulanin X6**	$C_{29}H_{37}N_5O_6$	552.2813	551.2743	*A. saccharicola*
	**Kojic acid**	$C_6O_4H_6$	143.0338	142.0265	*A. saccharicola and A. annui*
	**Phytosphingosine**	$C_{18}H_{39}NO_3$	318.3001	317.2928	*A. saccharicola and A. annui*
	**Chrysogine**	$C_{10}N_2O_2H_{10}$	191.0816	190.0743	*A. saccharicola*
	**Flavin**	$C_{12}H_{10}N_4O_2$	243.0874	242.0804	*A. annui*
	**Parasiticolide A (find by formula)**	$C_{26}O_8H_{30}$	493.1838	470.1946	*A. saccharicola*
	**Anthranilic acid**	$C_7H_7NO_2$	138.0550	137.0476	*A. saccharicola*
	**erythroglaucin**	$C_{16}H_{12}O_6$	301.0677	300.0634	*A. saccharicola*
	**Pantothenic acid**	C9H17NO5	220.1179	219.1106	*A. annui*

Top: the high-resolution MS/MS spectra of dereplicated metabolites; middle: the comparison between MS/MS spectra of certain metabolite and standard; bottom: the MS/MS spectra of standard compound in the in-house library.

Aflatoxin M1

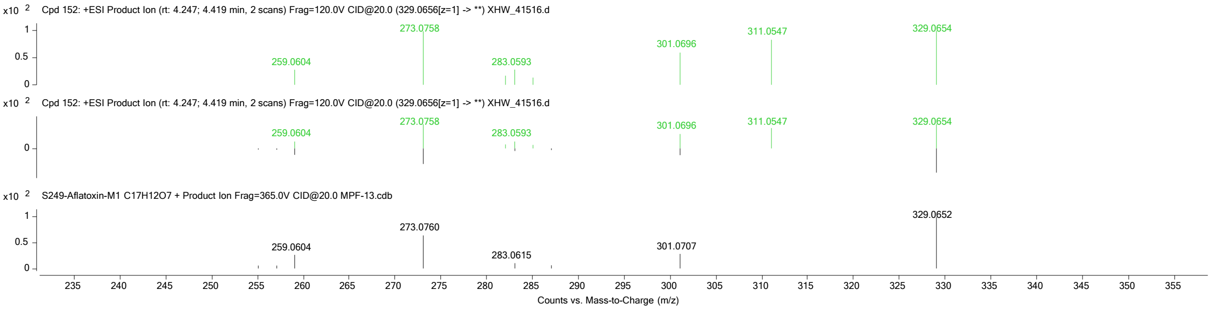

Aflatoxin G2

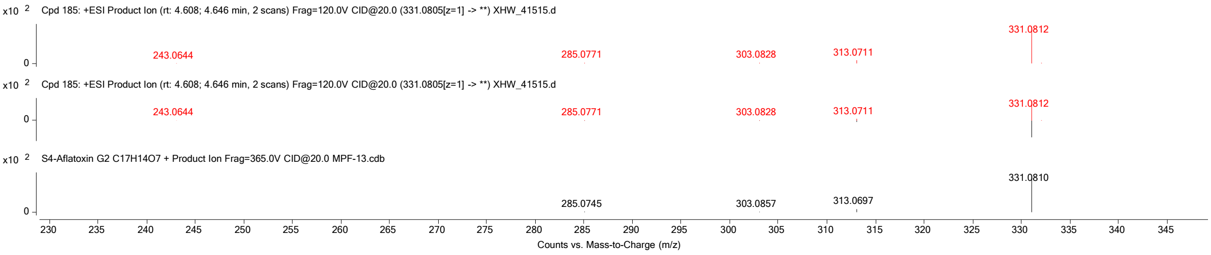

Aflatoxin G1

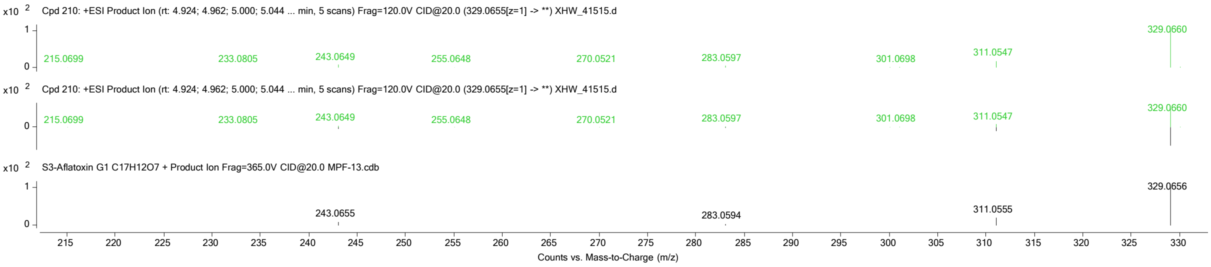

Aflatoxin B2

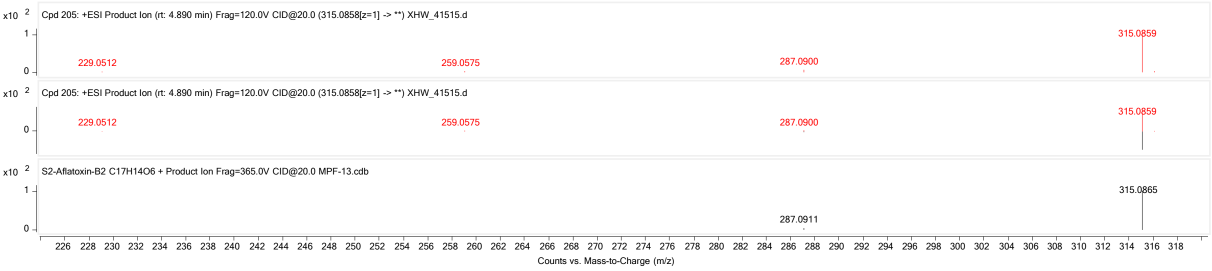

Aflatoxin B1

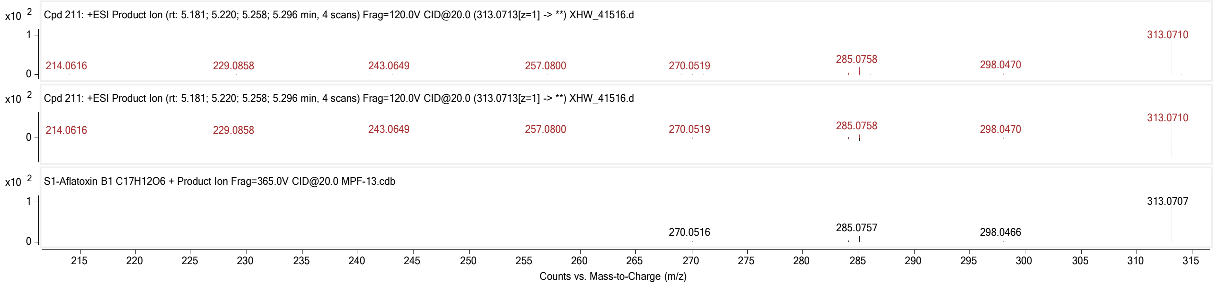

Sterigmatocystin

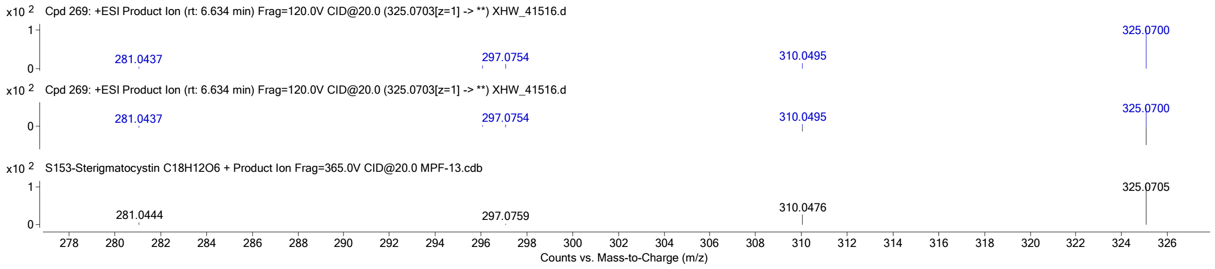

Cyclopiazonic acid

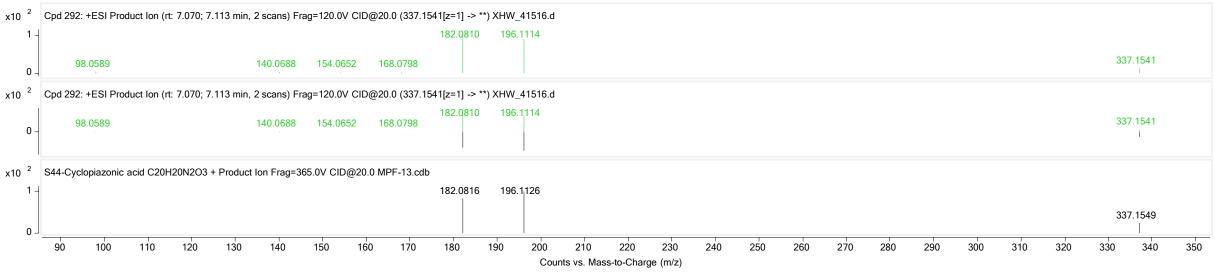

Valine-tenuazonic acid

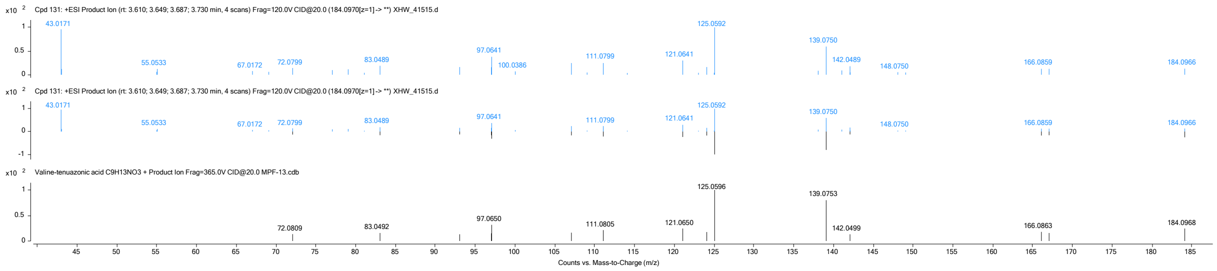

Tenuazonic acid

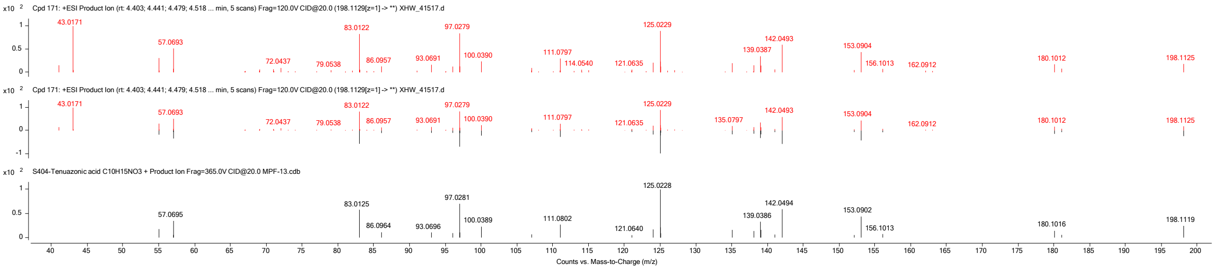

Desertorin A

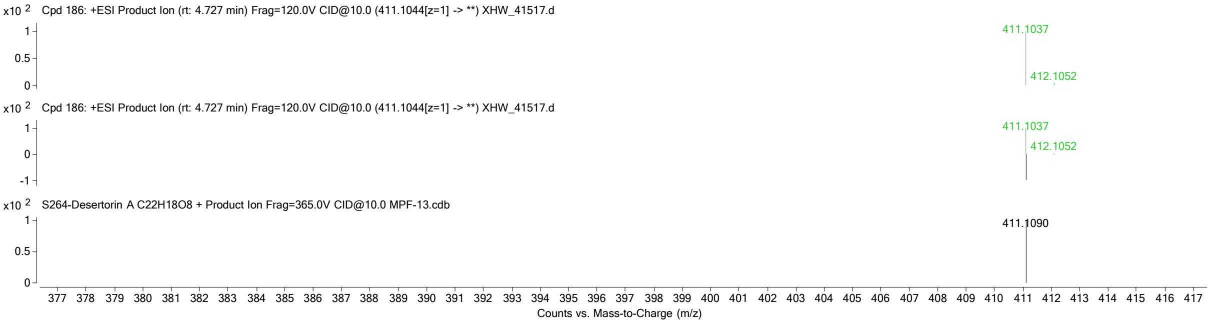

Kojic acid

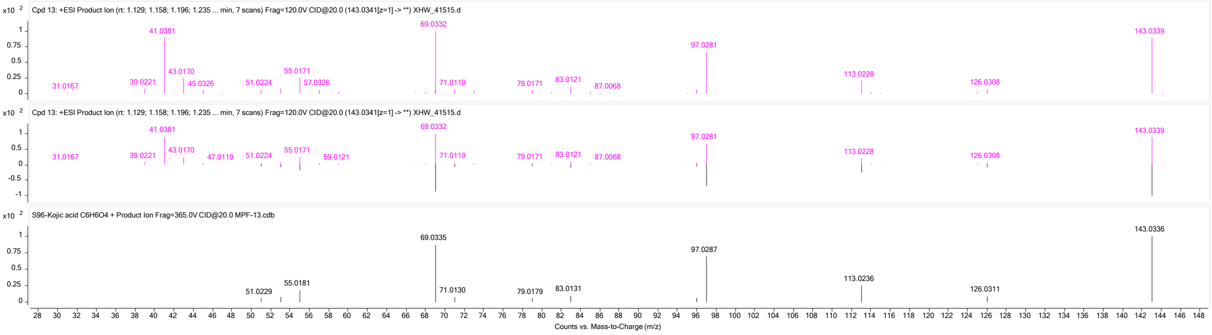

Nidulanin X3

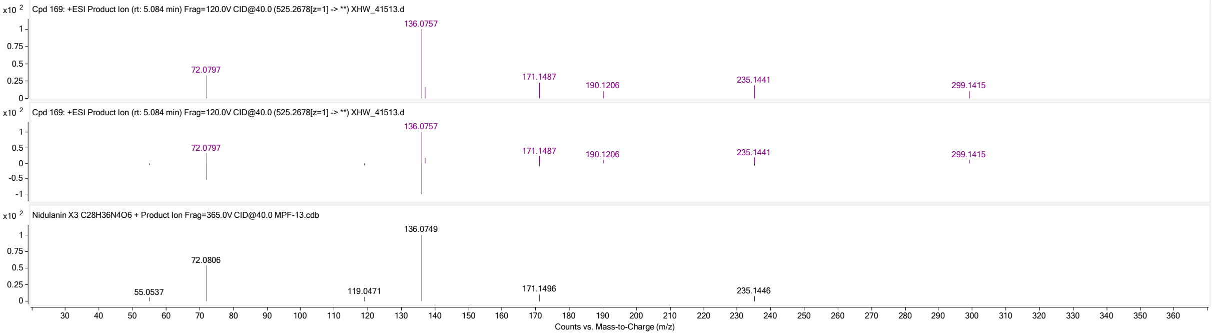

Nidulanin X5

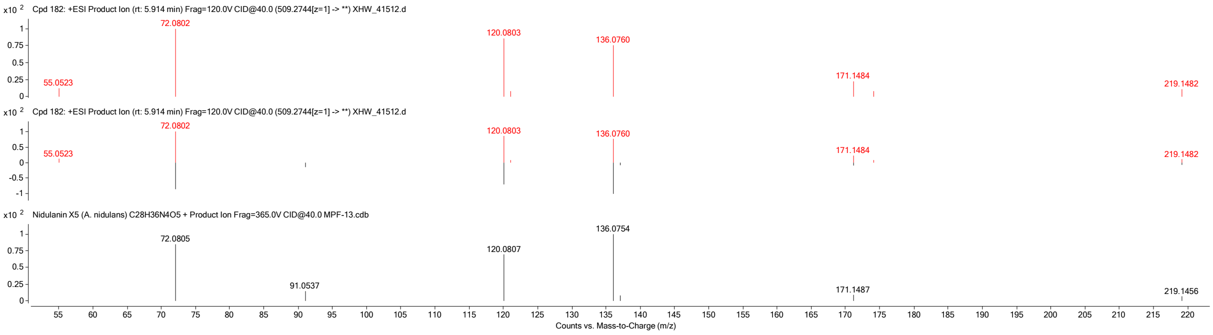

Nidulanin X6

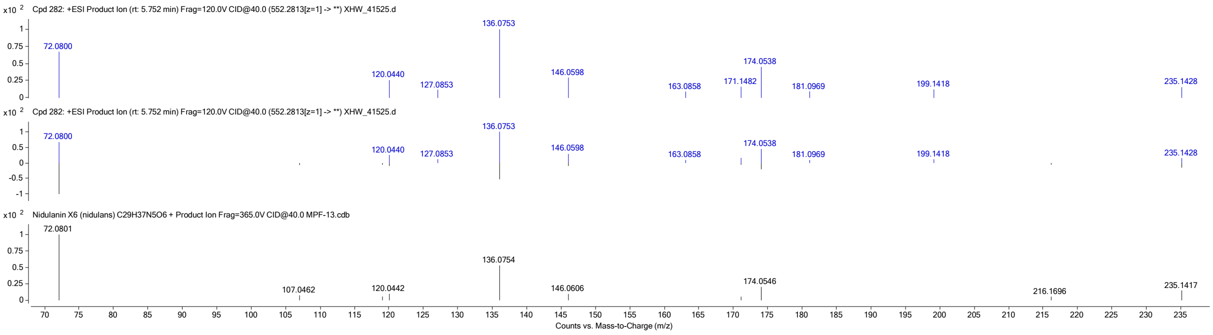

Anthranilic acid

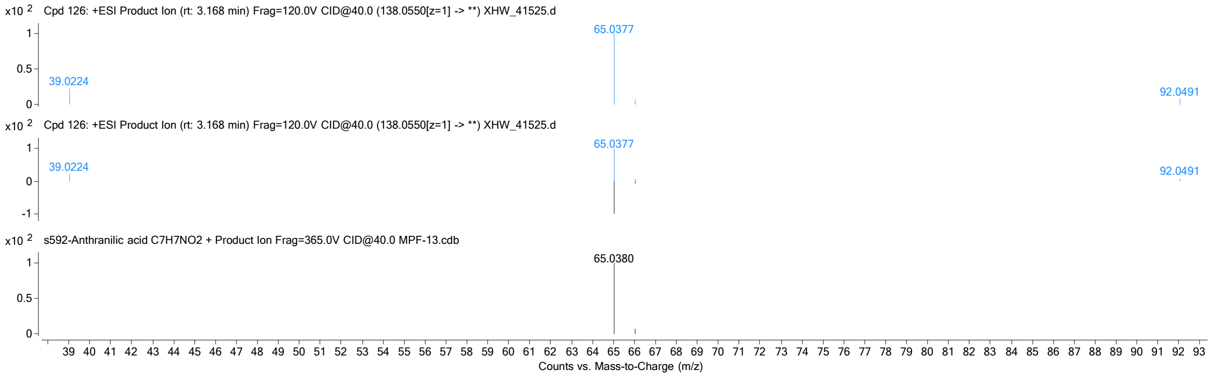

Pantothenic acid

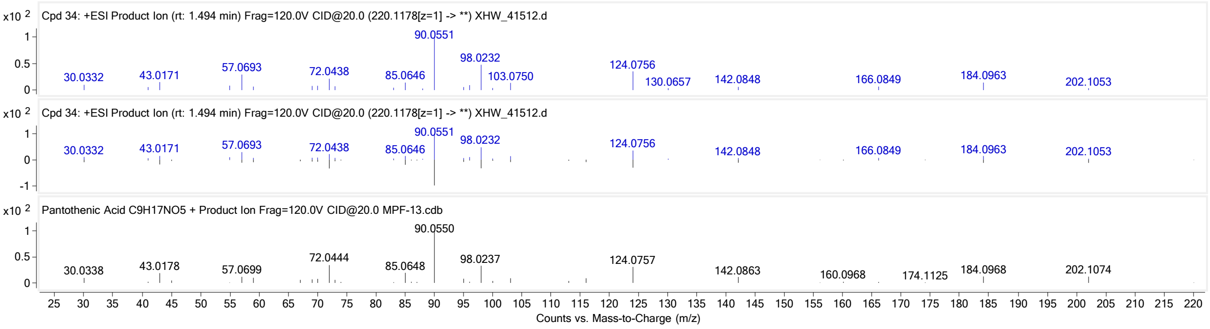

Erythroglaucin

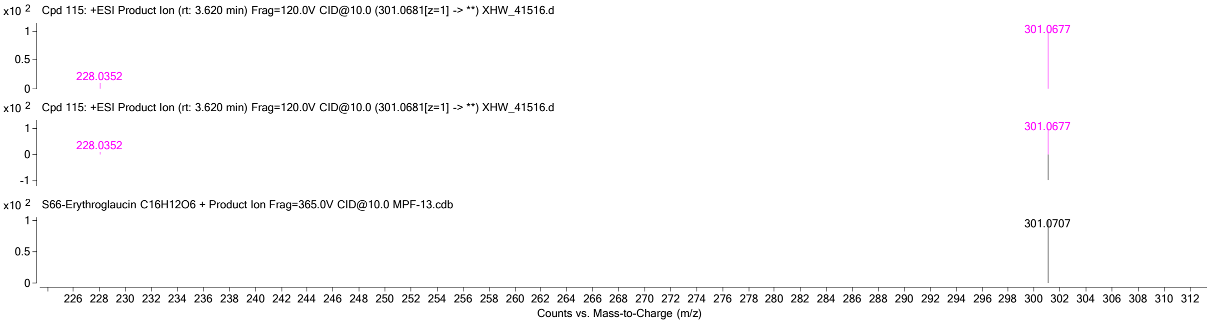

Aspergillic acid

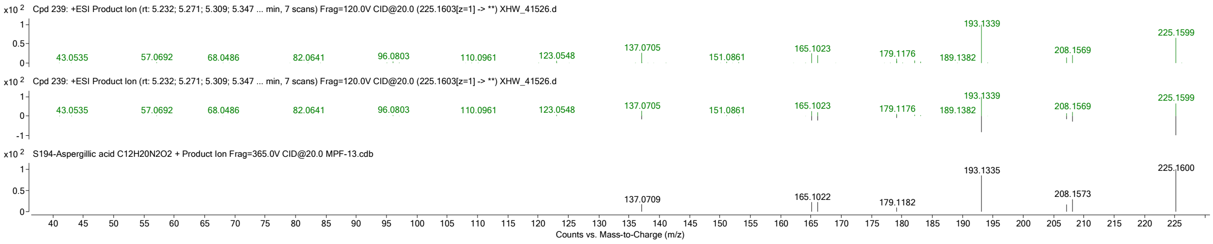

Chrysogine

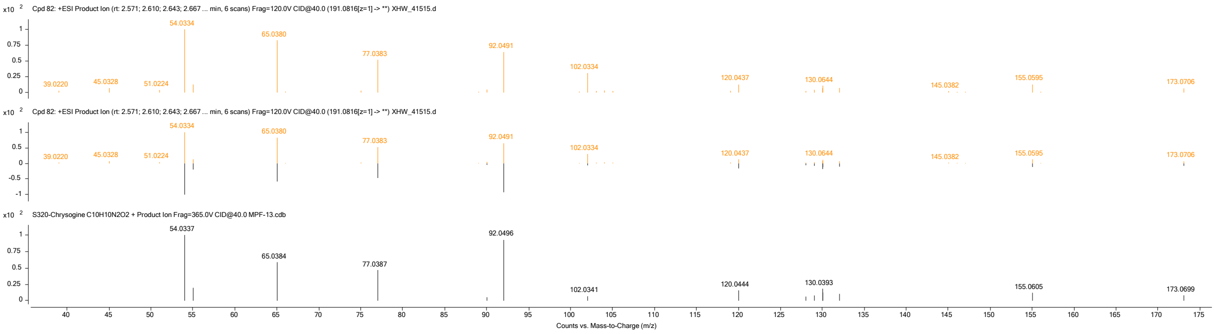

Flavin

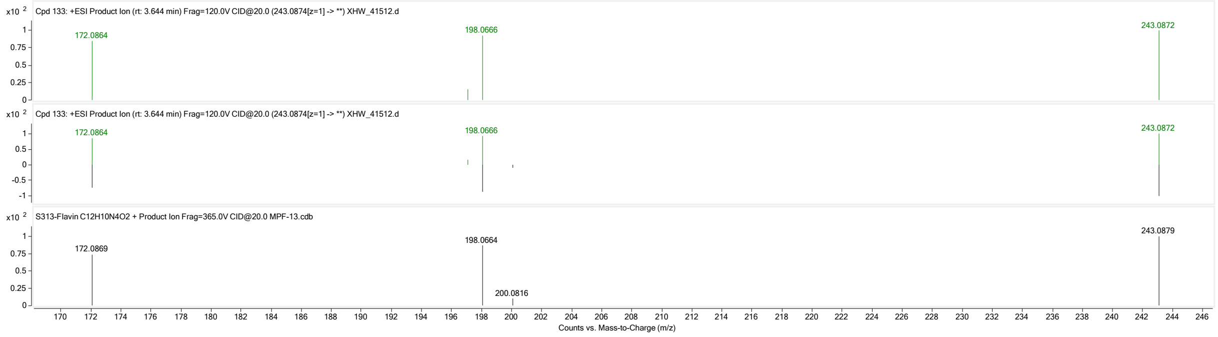

Ergokonin B

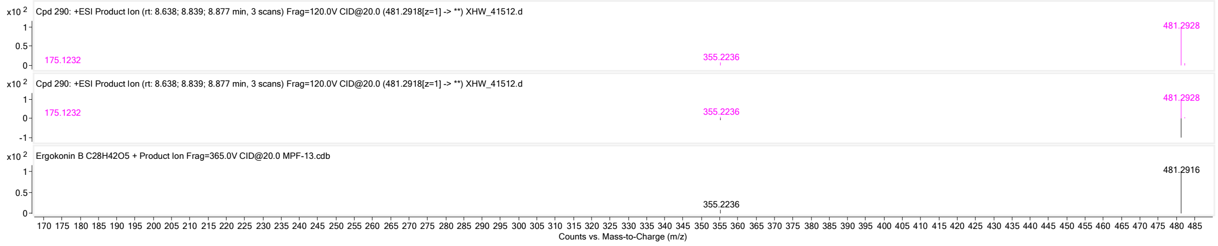

The HRESIMS (top) and the MS/MS (20 ev) spectra of parasiticolide A

Parasiticolide A (find by formula)

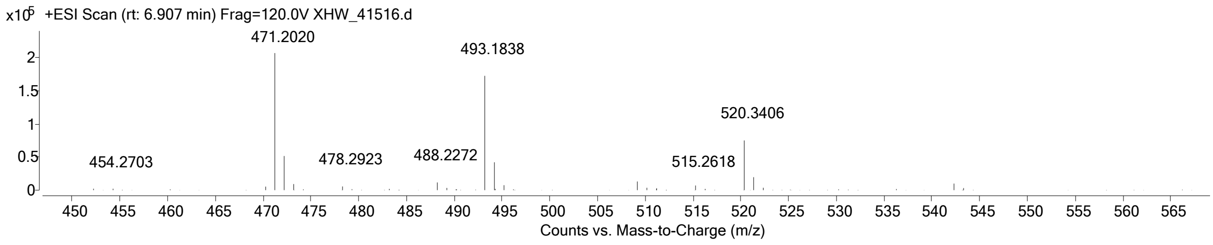

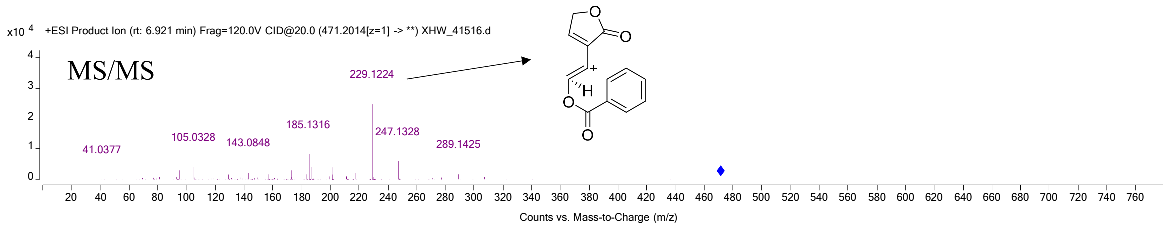

Supplement: Supplementary file 1 [file jof-08-01279-s001.zip › Table S3.pdf]
